# Spatial and Temporal Analysis of Water Quality in High Andean Lakes with Sentinel-2 Satellite Automatic Water Products

**DOI:** 10.3390/s23218774

**Published:** 2023-10-27

**Authors:** Johanna Elizabeth Ayala Izurieta, Andrés Agustín Beltrán Dávalos, Carlos Arturo Jara Santillán, Sofía Carolina Godoy Ponce, Shari Van Wittenberghe, Jochem Verrelst, Jesús Delegido

**Affiliations:** 1 Image Processing Laboratory (IPL), University of Valencia, 46980 Paterna, Spain; joeai@alumni.uv.es (J.E.A.I.); cjara@espoch.edu.ec (C.A.J.S.); Shari.Wittenberghe@uv.es (S.V.W.); jochem.verrelst@uv.es (J.V.); 2 Group of Research for Watershed Sustainability (GISOCH), Faculty of Sciences, Escuela Superior Politécnica de Chimborazo (ESPOCH), Riobamba 060155, Ecuador; abeltran@espoch.edu.ec (A.A.B.D.); sofia.godoy@espoch.edu.ec (S.C.G.P.); 3 Unit for Sustainable Environmental and Forest Management, Department of Soil Science and Agricultural Chemistry, University of Santiago de Compostela, E-27002 Lugo, Spain; 4 Research Group in the Natural Resources Field (GIARN), Faculty of Natural Resources, Escuela Superior Politécnica de Chimborazo (ESPOCH), Riobamba 060155, Ecuador

**Keywords:** Sentinel-2, atmospheric correction, satellite data, chlorophyll-a, water quality, lakes and lagoons, trophic state, C2RCC, total suspended solids, lake turbidity and transparency

## Abstract

The water of high Andean lakes is strongly affected by anthropic activities. However, due to its complexity this ecosystem is poorly researched. This study analyzes water quality using Sentinel-2 (S2) images in high Andean lakes with apparent different eutrophication states. Spatial and temporal patterns are assessed for biophysical water variables from automatic products as obtained from versions of C2RCC (Case 2 Regional Coast Color) processor (i.e., C2RCC, C2X, and C2X-COMPLEX) to observe water characteristics and eutrophication states in detail. These results were validated using in situ water sampling. C2X-COMPLEX appeared to be an appropriate option to study bodies of water with a complex dynamic of water composition. C2RCC was adequate for lakes with high transparency, typical for lakes of highlands with excellent water quality. The Yambo lake, with chlorophyll-a concentration (CHL) values of 79.6 ± 5 mg/m^3^, was in the eutrophic to hyper-eutrophic state. The Colta lake, with variable values of CHL, was between the oligotrophic to mesotrophic state, and the Atillo lakes, with values of 0.16 ± 0.1 mg/m^3^, were oligotrophic and even ultra-oligotrophic, which remained stable in the last few years. Automatic S2 water products give information about water quality, which in turn makes it possible to analyze its causes.

## 1. Introduction

Mountain regions cover 25% of the world’s land area and contain over 85% of the world’s amphibian, bird, and mammal species. The complex climatic characteristics are likely to play a key role in generating and maintaining diversity [1]. Mountainous regions, such as the high Andes, experience physical changes related to altitude (i.e., related to meters above sea level (m a.s.l.), such as atmospheric pressure, temperature, and clear-sky turbidity), as well as other changes, such as humidity, sunshine hours, wind, season length, geology, and even human land use [2]. Therefore, highland ecosystems and their dynamics are important in environmental, technological, social, and scientific interests. In addition, water regulation is one of the most important ecosystem services offered by high Andean ecosystems [3]. Water quality is vital for human populations, and the conservation of aquatic communities requires policies to prevent their deterioration and pollution [4]. However, these ecosystems are vulnerable and subject to spatial and temporal changes with negative impacts on the water quality [5].

The Andean mountain range of Ecuador contains about 25,000 shallow lakes with water bodies larger than 1 ha [6]. These ecosystems are located in páramo areas and foothills on high altitude plains where shallow lakes have formed [7]. Detailed information on their water status is currently absent. The region is characterized by complex geomorphology with limited access, and the presence of strong wind currents and wet–cold weather. As a result, in situ monitoring is time- and resource-consuming and is considered tedious, exhausting, and in some cases even impossible [8]. Therefore, methodologies using optical remote sensing technologies could serve as an alternative and attractive solution. Remote sensing imagery has been widely used for environmental and other studies [9,10], and has proven to be invaluable for water resource management at different scales [11,12].

Biophysical variables, such as chlorophyll-a concentration (CHL) and total suspended matter concentration, reveal changes in the water composition that can alter the optical properties. This can be analyzed using optical imagery due to differences in the shapes of the reflectance spectra associated with the dominant optical water quality constituents and relationships between water quality parameters such as Secchi depth and CHL [13]. These differences can be detected and quantified by satellite imagery. Furthermore, water bodies with high transparency are not the same as water bodies with high turbidity. Turbid waters require new processes for atmospheric correction; the backscatter ratio is spectrally neutral in small organic particles, and observations in turbid waters should take this into account [14,15]. Water has a low reflectance curve; atmospheric incidence reduces the radiance and accounts for the majority of the satellite-measured radiance in the visible bands [16,17]. Hence, the atmospheric correction process is an essential step in remote sensing studies of water quality [18]. Atmospheric corrections can be divided into either (1) absolute corrections, which result in surface reflectance and require atmospheric optical conditions (i.e., satellite image data with information on the corrections, independent data for atmospheric optical conditions, in situ data), and (2) relative corrections, which do not result in surface reflectance [19].

Regarding atmospheric correction, the Case 2 Regional CoastColour (C2RCC) is adaptable for various satellite instruments such as the Multi-Spectral Imager (MSI) from Sentinel-2 (S2), making an approach generic across missions [20]. This processor makes it possible to retrieve the reflected light spectrum over a water body [18]. The Case 2 Regional CoastColour (C2RCC) processor is based on a large database of simulated water-leaving reflectances and the related top-of-atmosphere radiance with the purpose of obtaining the water radiance and retrieving the inherent optical properties of the water body [18,21]. The database is used to train a neural network model obtaining the inversion of the spectrum for the atmospheric correction [22]. Part of the Sentinel fleet, as managed by the European Space Agency (ESA), includes the S2 mission with MSI sensor. Since 2016, the S2 constellation has offered high spatial (10–20 m) and temporal resolution (5 days), with free access [23]. S2 imagery can be applied to water quality monitoring [24], for instance for extracting automatic products for water. The atmospheric correction model C2RCC has been adapted to S2. Three versions are available in C2RCC, i.e., C2RCC, C2X, and C2X-COMPLEX [25]. These different versions make it possible to study lakes and lagoons with different eutrophic states with the estimation and evaluation of water variables such as CHL, solids in suspension, and transparency.

Given the complexity of mountainous ecosystems and the atmospheric effects impacting remote sensing products for water studies, in situ sampling is required to validate or detect problems and errors related to particular conditions over the study zones. With the ambition of evaluating the performance of the C2RCC automatic products using S2 imagery, we analyzed three lakes in the highlands over the Ecuadorian Andean Mountain range with different eutrophication states. Within this context, the objectives of this study are twofold: first, to validate biophysical variables obtained from three C2RCC atmospheric correction versions using in situ samples and acquired S2 images on three lakes with different trophic statuses; and second, to analyze spatial and temporal changes in water composition for the study lakes and their link with environmental processes.

## 2. Materials and Methods

### 2.1. Study Area

**Figure 1:**
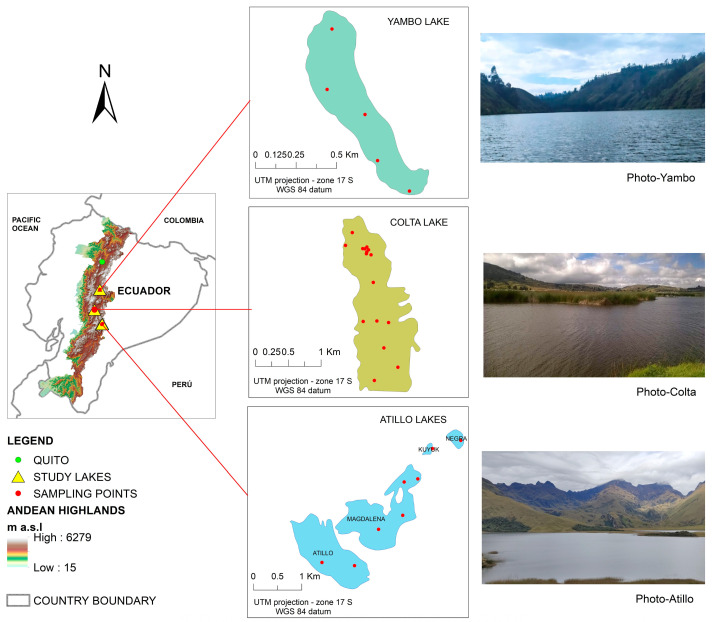
High Andean lakes studied and the distribution of sampling points; CHL in Yambo and Atillo and transparency using the SECCHI disk depth (SD) in Colta.

In Ecuador, the Andean mountain range runs the country from north–south through 10 of its 24 provinces, from Carchi to Loja. Here, around 90 percent of the lakes and water bodies are distributed above 3000 m a.s.l. This study focused on three high Andean lakes of the center zone of Ecuador, i.e., the Yambo, Colta, and Atillo lakes (see Figure 1).

The Yambo lake is located in Cotopaxi province. The lake encompasses an area of 25 ha approximately at 2600 m a.s.l. located at 78°59′ W and 1°10′ S. The annual average of temperature in the last five years was 11 °C [26]. A tourist facility is located in the southern area of Yambo lake. Also, tourism activities are developed on Yambo lake mainly in the northeast zone with a port for recreational activities. Therefore, socioeconomic activities take place in the surroundings. Agricultural activities introduce excreta, and its accumulation through nitrification processes in the water can cause the contamination of water bodies. All the activities around the lake and the resulting discharges do not maintain specialized treatment in order to minimize a negative environmental impact on their waters. Consequently, these activities can generate eutrophication impacts related to changes in the compositional characteristics of water and also its quality. With a visible greenish coloration, the water of the lakes shows the presence of increases in phytoplankton and a high concentration of plant biomass.

Similarly, located in the Chimborazo province at 3320 m a.s.l., Colta is also a touristic lake. Colta has an area of approximately 186 ha situated at 78°12′ W and 1° S. The lake is located next to the town center and the Pan-American highway; therefore, its north zone is easily accessible. Hence, anthropic activities derived from tourism, agriculture, livestock, fumigation, and wastewater discharges have a negative impact on the lake ecosystem. Main and secondary streams possibly introduce large amounts of sediment into the lake due to hydric erosion processes [27]. Due to public interest in lake recovery, the lake has been dredged on irregular dates as a mechanism for control and management of Colta water since 2011 [28]. Also, the lake and its surroundings are affected due to poor water residual management, and the increase in the agricultural area produces negative environmental impacts. The resident species can influence biophysical water parameters due to a great variety of birds of around 20 species and 13 families, with the families Anatidae and Tyrannidae being the most abundant [29].

Finally, Atillo is located in the southeastern zone of the Chimborazo province and extends between 78°52′ W and 2°19′ S. This lacustrine ecosystem is formed by four main lakes (i.e., Atillo, Magdalena, Kuyuk, and Negra). Atillo at 3440 m a.s.l and Magdalena at 3445 m a.s.l. are the largest lakes, with an area of 125 and 133 ha, respectively. Kuyuk with an area of 2.8 ha is an affluent area of Magdalena. The water coloration of these lakes is of blue-green color, unlike Negra. The Negra Lake has an area of 8.8 ha and it is located at 3450 m a.s.l.; its water has dark coloring (varies between black, green, and blueish depending on the weather conditions); and it is surrounded by cliffs and large extensions of grasslands. Currently, this lacustrine ecosystem is characterized by low anthropic activities and high quality water. The soil water retention is over 50 percent [30]. The lower temperatures limit algae growth, increasing the water transparency, and variables such as CHL and temperature can alter the spectral phytoplankton absorption coefficient [31].

### 2.2. Water Sampling

Due to the extreme conditions of these lakes and the weather conditions of these zones nearest to zero latitude (equatorial line), the seasons are not the same as in other latitudes. Therefore, the planification focused on a range of days with apparently better weather conditions. The water sampling was carried out on multiple dates, and samples were obtained during a day per each month and were planned to consider the climatic conditions in the last week of the month. Yambo was monitored from January to March of 2021, and five water samples were collected for each month at the same UTM coordinates; Colta during was sampled from April until June of 2020; and Atillo was sampled during November 2020 until January 2021. The water sampling distribution was obtained taking into account the spatial representativity, access conditions, and avoiding the lakes’ boundaries (see Figure 1). Finally, 5 sampling points were used for the Yambo lake, 14 in Colta, and 8 sampling points in Atillo.

The procedure for water sampling at each location was developed by a team of Group of Research for Watershed Sustainability-GISOCH, using a rowing boat to move from one sampling point to another. The methodology for obtaining water samples was based on the national standard technical note [32]. The process consisted of collecting 750 mL of water at different depths, considering the maximum depth and Secchi disk readings using the standard [33]. Surface water was collected using a graduated collector and poured into previously coded and labeled glass bottles. Samples at depth were obtained using the Van Dorn sampler, which is introduced into the lake attached to a numbered rope every two meters, and then the water was placed in the graduated collector and finally deposited carefully into the glass bottles. The samples were stored in coolers with ice to maintain the temperature up to their corresponding transportation to the Water Quality Laboratory, complying with the norm for the conservation of water samples [34].

The chlorophyll concentration values for each sampling point were extracted in the laboratory using spectrophotometer filtration equipment from Thermo Scientific (Thermo scientific evolution 201 UV-Visible, Madison, WI, USA) with the Standard Methods 10200 H [35] (Equation (1)). Water samples of 200 mL were filtered with 0.45 µm cellulose nitrate filters, and then the filters were inserted into 15 mL Falcon tubes. The spectrophotometer was set up and calibrated with 90% acetone as a blank. Each prepared sample is then processed, obtaining absorbance values of 750 and 664 nm for each sample. Finally the CHL is obtained using Equation (1), where V_1_ is the extracted volume [L]; V_2_ is the sample volume [m^3^]; L is the light path or width of the spectrophotometric cell [cm]; 26.7 is a constant value representing the absorbance correction factor. CHL is obtained from the turbidity corrected 664 nm before acidification and 665 nm after acidification readings by subtracting the 750 nm of absorption reading from its respective 664 nm and 665 nm readings:

(1) $$CHL\left(\frac{mg}{m^{3}}\right)=\frac{26.7\times {(}_{corr.664-corr.665})\times V_{1}}{V_{2}\times L}$$

### 2.3. Sentinel 2 Data and Processing and Atmospheric Correction

This study used multispectral images from Sentinel-2 (S2) mission satellites. The spatial resolution with bands of S2 (i.e., 10 m, 20 m, and 60 m) allows for improving the ability to analyze smaller areas of the earth’s surface and also to obtain images with a temporal resolution of up to 5 days with S2-A and S2-B satellites. Level-1C products were used and downloaded from the Copernicus Open Access Hub. The Hub is a web service from Copernicus Program of the European Union and ESA [23], providing top-of-atmosphere (TOA) reflectances with all parameters to transform them into radiances [36] for the study zone. The low temperatures over this ecosystem facilitate a lack of thermal seasonality, especially with the presence of the predominant convective and orographic cloud formations [37]. Hence, cloudiness cover (CC) and cloud shadows are a constant problem when using optical sensors on high mountain ecosystems [8]; their higher percentages over scenes reduce the amount of images with high quality that can be obtained. Similar Andean zones show low climatic variability in a year. For instance, the mean annual temperature in the year 2021 in the high Ecuadorian Andean mountains ranges from 9 to 15 °C [26]. Therefore, the images used corresponded to the acquisition date closest to the sampling period, in the same sampling month [38,39]. Tiles 17MQU and 17 MQT with scenes of 100 × 100 km^2^ were used. The used satellite imagery is detailed in Table 1.

Optical satellite data applied for water monitoring studies require a specific atmospheric correction due to the low reflectances at the water surface. Hence, the signal measured at the satellite is influenced at around 90% by the atmospheric path radiance [18]. For the estimation of the constituents of water or its optical properties, the inversion of the spectra of the emergent reflectance of the water is required, so all images were processed using the C2RCC (Case 2 Regional Coast Color) processor of the toolbox for thematic water processing using the Sentinel Applications Platform (SNAP) software version 9.0.0 [25]. This process obtains water variables from the inversion of a database of radiative transfer simulations and neural networks, which can be applied to a diverse number of sensors. The C2RCC processor relies on a large database of simulated water-leaving reflectances, and includes the related top-of-atmosphere radiances that were used to train neural networks (NN). The NN then produces the inversion of the spectrum and uses it for the atmospheric correction process. As a result, the determination of the water surface reflectance is obtained, as well as the retrieval of the inherent optical properties of the water body for pigment absorption, detritus, gelbstoff, and total scattering [18].

C2RCC offers three versions in the latest SNAP releases providing automatic water quality products; each version uses pre-defined sets of NN, which differ in the training ranges of the inherent optical properties (IOPs) [40]: (1) the C2RCC version is the original network covering typical ranges of coastal IOPs, which has applications for waters with low turbidity such as marine and ocean waters; also (2) the C2X networks use a CoastColour dataset to extend the range for coastal waters, including extreme cases [18], and it is used for studies in inland waters with high concentrations of suspended material and CHL; and (3) C2X-COMPLEX was trained with intermediate ranges of IOPs [41], and it is used for darker waters (i.e., optically complex water types, preferably to be used for inland waters) [25,42].

When applying the atmospheric correction with each of the three versions, in addition to the retrieval at the water surface reflectance, a number of automatic products are generated in SNAP. Among them, the one that best defines transparency is Kd_Z90max, which corresponds to the depth (in m) at which 90% of the radiation reaching the water surface is absorbed. Kd_Z90max comes from (1/kdmin), where kdmin is the mean irradiance attenuation coefficient at the three bands with minimum kd in [m^−1^] [4,5]. The automatic products like CHL and total suspended matter concentration (TSM) were obtained with the default factors and exponents of C2RCC (CHL = 21 × a_pig^1.04^; TSM = 1.72 Palatino Linotype b_part + b_wit Palatino Linotype 3.1) [40], where a_pig, b_part and b_wit are optical properties of water at 443 nm wavelengths, i.e., a_pig is the absorption coefficient of phytoplankton pigments, b_part is the scattering coefficient of typical sediments, and b_wit is the scattering coefficient of white particles (calcareous sediments) [40]. As a result, by applying atmospheric correction with each one of the three versions, absolute concentrations of CHL in [mg/m^3^], TSM in [g/m^3^], and Kd_Z90max in [m] are obtained [25].

**Figure 2:**
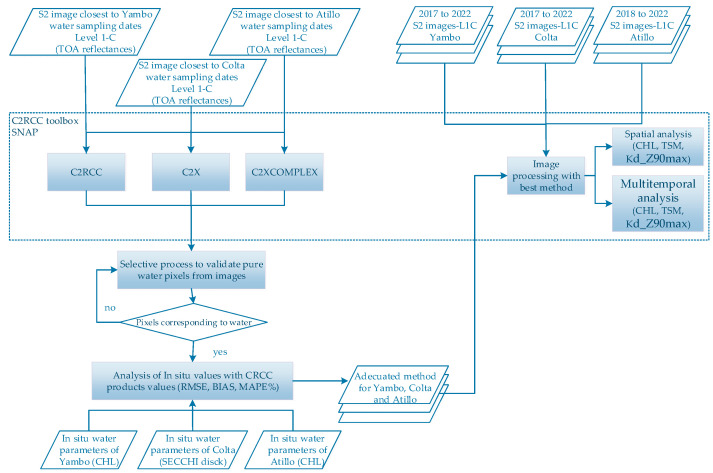
Processes diagram for image processing.

A selective process to validate pixels was made using UTM coordinates from in situ data sampling and by verifying that the spectral signature observed is water (see Figure 2). Due to the narrow shape of the north zone of the Colta lake, it was necessary to go through the pixel selection to the next closest pixel of water. Subsequently, in order to determine the best C2RCC version to apply in each of the three studied lakes and due to the singular characteristics of each study lake explained in Section 2.1, the three automatic products generated by each version were evaluated by comparing these with the field data (see Figure 2). In the Yambo and Atillo lakes, this analysis was achieved by comparing the automatic CHL product, obtained for images corresponding to dates closest to the sampling date, with the field CHL data. Information from the satellite image product was used based on pixels corresponding to GPS position from in situ samples and avoiding outliers. In Colta, CHL data were not available, so sampling was performed by comparing the Secchi disk depth (SD in m) with the automatic Kd_Z90max product, as they are comparable variables of similar rank [40,42], and also Kd_Z90max is a variable strongly correlated with the SD [43,44]. The lowest root mean square error (RMSE) (Equation (2)) value (results of the comparison of each method’s products with in situ data variables) determines the most applicable atmospheric correction method over the bodies of water, and is subsequently used for multitemporal analysis for each lacustrine ecosystem. Additional statistical indicators are as follows: the Bias (Equation (3)) and Mean Absolute Percentage Error (MAPE%) (Equation (4)) were obtained to compare values estimated with the measured values.

(2) $$RMSE=\sqrt{\sum_{i=1}^{N}{\frac{\left({X_{i}}^{estimated}-{X_{i}}^{measured}\right)}{N}}^{2}}$$

(3) $$BIAS=\frac{1}{N}\sum_{i=1}^{N}{{(X}_{i}}^{estimated}-{X_{i}}^{measured})$$

(4) $$MAPE\%=\frac{100}{N}\sum_{i=1}^{N}\frac{\left|{X_{i}}^{estimated}-{X_{i}}^{measured}\right|}{{X_{i}}^{measured}}$$

Once the most accurate C2RCC version had been determined for each lake, the lacustrine ecosystems were statistically analyzed in two ways: (1) spatial analysis and (2) multitemporal study. For spatial variability, distributions of the automatic products CHL, TSM, and Kd_Z90max were retrieved for one image. For the multitemporal study, the mean and standard deviation of each of the three variables in a region of interest (ROI) in each lake were calculated on different dates (from 2017 to 2022 for Yambo and Colta, and from 2018 to 2022 for Atillo), as shown in Table 1. Due to the cloudy conditions of the study area, careful manual delimitation of ROIs for each lake was necessary. The ROIs were generated using the S2-mask cover products, and by using visual interpretation, avoiding the edges of lakes. The number of required ROIs depends on the image and conditions of the study area to ensure the quality of pixels/pure water pixels without interference from soil or clouds (i.e., islets or edge land, fog, and clouds) and avoiding adjacency effects.

**Figure 3:**
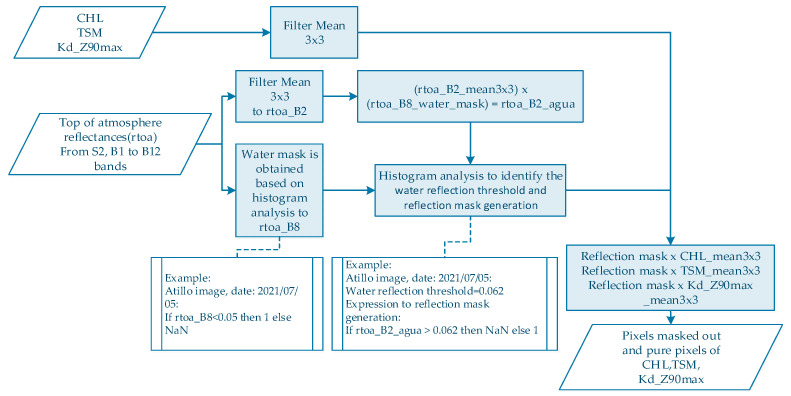
Process diagram for masked-out pixels due to reflections caused by waves come from wind.

Subsequently, C2RCC-methods do not include specific correction for sun glint or land adjacency [40]. Hence, due to an effect of wind over water surface that alters the signal retrieval, an additional process of encapsulation was applied based on histogram analysis over S2-bands. Therefore, all pixels with reflections caused by waves that come from wind were masked out (see Figure 3).

Then, all variable values were obtained with the accumulative ROIs for each lake. As a result, these variables could expose the water quality in a multitemporal study. Subsequently, an environmental analysis was carried out to evaluate the trophic state of the lakes. Social activities in the lakes’ surroundings and weather variables were also evaluated to better understand the lakes’ conditions. This information could also be used to detect sources of discharge.

## 3. Results

### 3.1. Atmospheric Correction Method for Yambo, Colta, and Atillo Lakes

**Figure 4:**
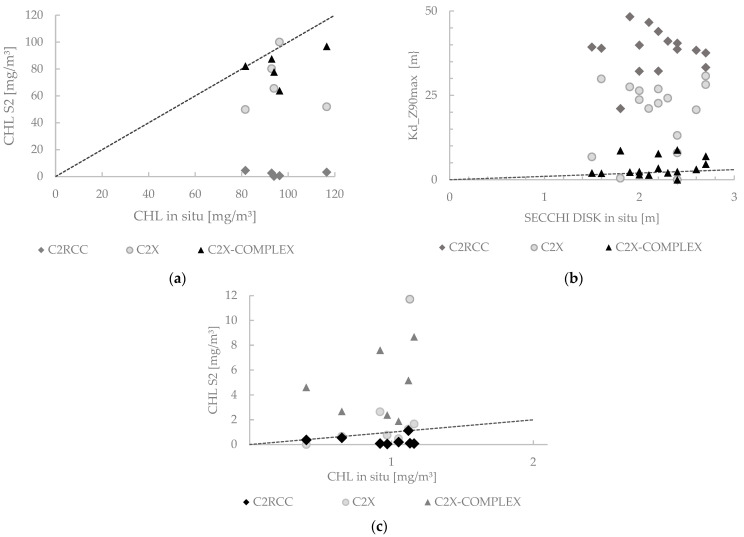
Scatterplots between the in situ sampled data and the retrieved data by the different atmospheric correction C2RCC versions: CHL for Yambo (**a**), Secchi disk vs. Kd_Z90max in Colta (**b**), and CHL in Atillo lake (**c**). The 1:1-line is added.

RMSE, BIAS, and MAPE% values as calculated by field data against the values from the automatic products of each C2RCC version are given in Table 2. Results are in agreement with the physical characteristics of the lakes. Yambo and Colta have a higher degree of anthropic intervention and are more turbid, while Atillo is a lake with low intervention and superior quality. Based on the lowest RMSE, BIAS, and MAPE% values, the best atmospheric correction C2RCC version for Yambo lake was C2X-COMPLEX (see Table 2). Yambo has a simple shape which favors a reduction in spatial resolution. In the Colta lake, the C2X-COMPLEX also gave the lowest RMSE value. Colta has an elongated shape with very narrow sectors, especially in the northern part. The results revealed the C2X-COMPLEX as the most adequate version for Colta. Finally, the results indicate that for Atillo the most adequate atmospheric correction C2RCC version was C2RCC. These results also can be observed in Figure 4, which shows scatterplots between in situ sampled data (i.e., CHL for Yambo, SD for Colta, and CHL for Atillo) and data retrieved by the C2RCC, C2X, and C2X-COMPLEX versions. Please refer to the Supplementary Materials.

### 3.2. Spatial Analysis

**Figure 5:**
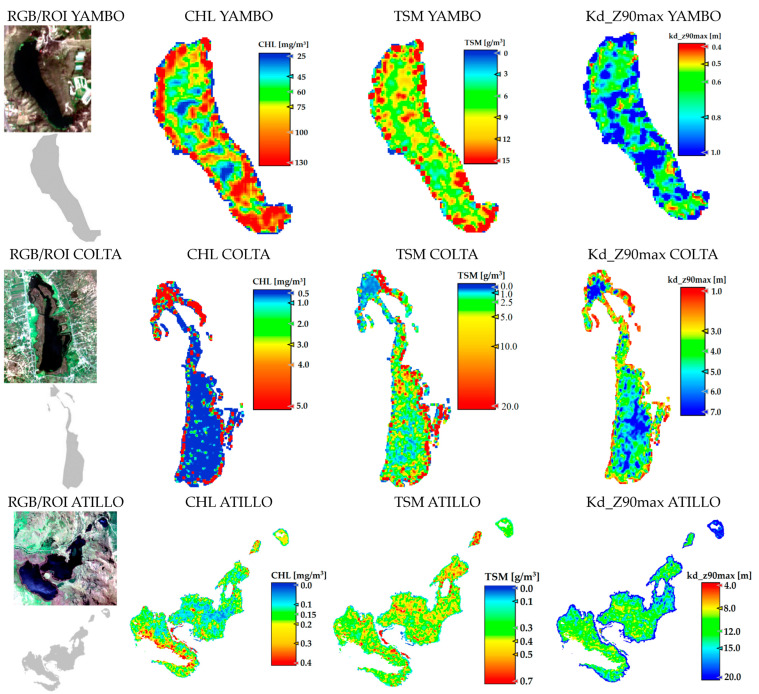
Spatial distribution of CHL, TSM, and Kd_Z90max using the best atmospheric correction C2RCC version for each lake. RGB and ROIs of satellite images are shown. Image dates are shown on Table 3.

The spatial distribution of CHL, TSM, and Kd_Z90max based on the best atmospheric correction method for each lake is shown in Figure 5. Using S2 images approximating most closely the sampling dates and based on ROIs to obtain variables of pure water pixels, the results indicate that Yambo obtained the highest CHL values with a mean of 79.6 mg/m^3^, followed by Colta with 0.5 mg/m^3^, and finally Atillo with 0.16 mg/m^3^ (see Table 3). The north sector of Colta presents differences in CHL values compared to the other lakes. As for Atillo, it has a homogenous distribution.

Regarding the TSM values, Yambo also obtained the highest mean value, followed by Colta and Atillo, with TSM mean values of 9.2 g/m^3^, 4.4 g/m^3^, and 0.38 g/m^3^, respectively. The spatial distribution of TSM is mainly homogeneous except for the northern sector of Colta. Kd_Z90max values show an inverse behavior with higher values on Atillo and lower values in Yambo; considering only ROIs, the extended lakes show homogeneous spatial distribution. The southern area of Atillo shows an area with very high anomalous values of CHL and TSM. This may be due to reflections caused by strong winds (see also the Atillo RGB image: Figure 5).

Yambo is located in a valley at 2600 m a.s.l. and the west and east center zones are rounded by median slopes between 20 and 30 degrees, whose highest points are approximately 170 m above the level of the lake surface. Hence, discharges could be linked with the dissolution of minerals by soil erosion around the lake and the geological composition. In addition, a Pan-American highway is located at the top near to the northwest zone with a tourist viewpoint; also in the northeast zone a tourist port is placed, and a tourist facility is located in the southern zone. These land uses can be a source of residual discharges to lakes that would cause negative environmental impact due to associated human activities [33]. Hence, different types of waste are directly discharged into the lake. This can be tracked by higher CHL and TSM values observed in the northeast and south zones of Yambo. The depth of the Yambo lake is approximately 25 m, but lower Kd_Z90max values ranging from 0.5 to 1 m imply that sunlight can penetrate into the first meter of water depth. In addition, the higher values are correlated with sectors with lower CHL and TSM values.

Like Yambo, the Colta lake corresponds to volcanic origin. Colta is characterized by high tourist activities; part of this is due to its proximity to two roads localized around all sides of the lake. Also, agricultural activities and the town center are nearby. In the northwest zone the highest CHL values were observed in the narrowest zone. A stream flow analysis around Colta indicated major discharges in the north zone, which seems to reveal that human and agricultural activities generate discharges into springs and streams. Hence, they may carry nitrites to the lake, producing the high CHL shown. In addition, a boardwalk is located 30 m from the highway and up to 40 m from the lake, and also a touristic activities area was developed on this zone of the lake. TSM variability was observed on the south sector of Colta; it would be related to around 21 streams that generate water erosion and drag sediments. The depth is variable with a mean of 3.5 m [45], depending on rainfall and seasonal streams.

Low values of CHL and TSM are observed in the Atillo lakes even with a road emplaced a few meters along the lakes, which can be explained by the fact that Atillo is located in a protected natural area. Even so, it is known that one of the activities that is carried out in Atillo is fishing. Although the climatic conditions suggest having similar radiation temperatures throughout the year, the inclusion of winds and rain tends to increase between January to April, which may contribute to thermal stratification [46]. This phenomenon would be relatively low as opposed to tropical environments with seasonal periods, but even so, it can be linked with phytoplankton migration to another strata in the water column.

Higher Kd_Z90max values are related to lower CHL and TSM values (see Table 3). In Atillo, the range of CHL and TSM is low and this group of lakes at 3440 m a.s.l are the highest in comparison to Colta and Yambo. The climatic conditions in this lake located in a protected area, among others factors, would have an effect over the growth of algae and therefore the transparency. An additional factor should be taken into account over the Atillo analysis: the water properties added to the wind conditions of the area alter the results. Therefore, the water seems to generate reflections due to the effect of waves caused by the wind over its surface (see Figure 5). Hence, the ROI used for statistical analysis was reduced for Atillo.

### 3.3. Multitemporal Analysis

**Figure 6:**
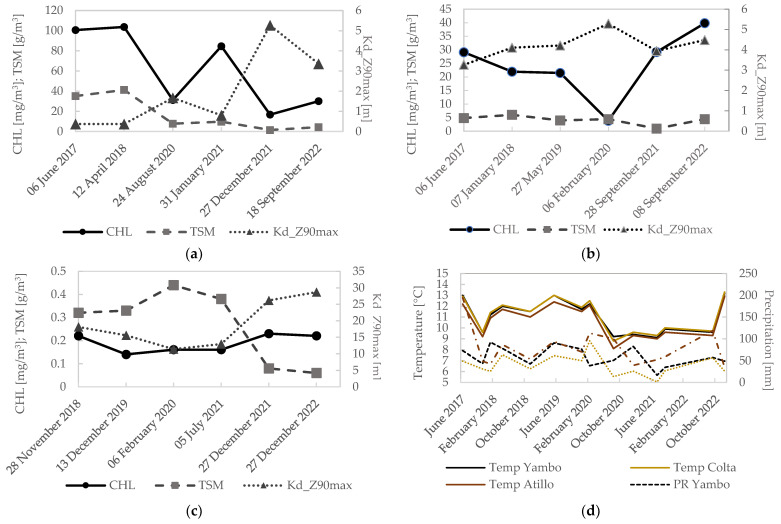
Multitemporal variability (2017–2020) for pure water pixels using ROIs from automatic water products: (**a**) Yambo lake; (**b**) Colta lake; (**c**) Atillo lakes. Kd_Z90max values are on the secondary axis. (**d**) Temperature and precipitation variability for Yambo, Colta, and Atillo lakes [47,49].

**Figure 7:**
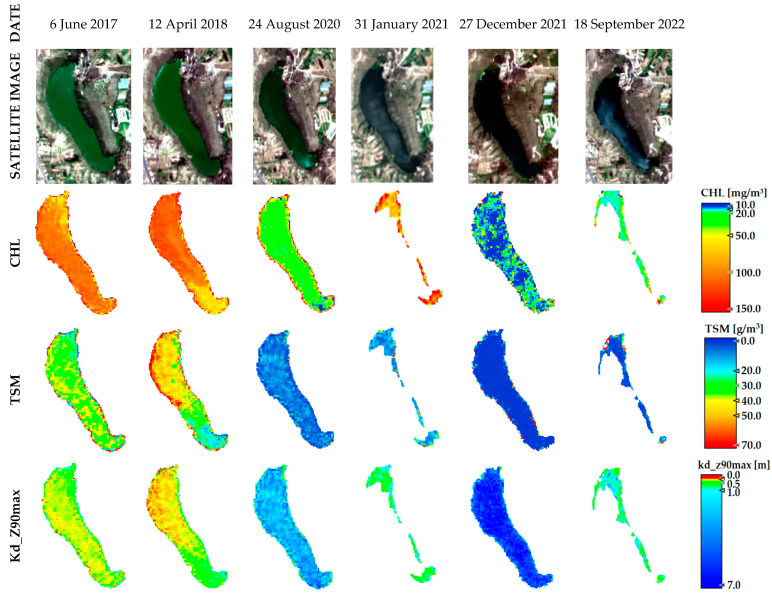
Multitemporal variability of CHL, TSM, and Kd_Z90max in Yambo lake from 2017 to 2022 years.

The multitemporal CHL, TSM, and Kd_Z90max results (mean values in each ROI) for each lake from 2017 to 2022 for Yambo and Colta, and from 2018 to 2022 for Atillo, are shown in Figure 6. Yambo shows high values of CHL; however, notable decreases are shown in 2020, at the end of 2021, and in September 2022. The temperature values obtained from climate weather monitoring stations [20,35] and precipitation using the Climate Hazards Group InfraRed Precipitation with Stations (CHIRPS) data [47,48] (see Figure 6d) observed for Yambo in during the multitemporal analysis could indicate a certain linking with the reduction in CHL values, which is noticeable for evaluation dates in 2021 and 2022 (see Figure 7). A decrease in TSM values is also observed from 2019 onwards. In addition, the results of TSM allowed us to detect a discharge of solids in the northern sector of the lake, which is in concordance with anthropic activities due to the port. This discharge was also detected by the decrease in Kd_Z90max values (see Figure 7). Regarding Kd_Z90max in Yambo, the results suggest that in 2021 and 2022, the water transparency was higher due to the fact that CHL and TSM values were lower.

**Figure 8:**
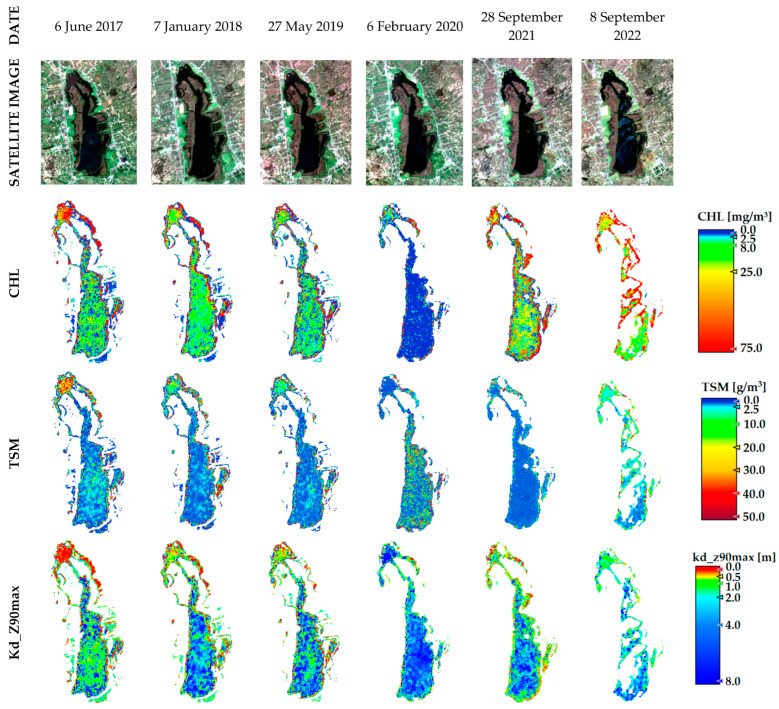
Multitemporal variability of CHL, TSM, and Kd_Z90max in Colta lake from 2017 to 2022 years.

**Figure 9:**
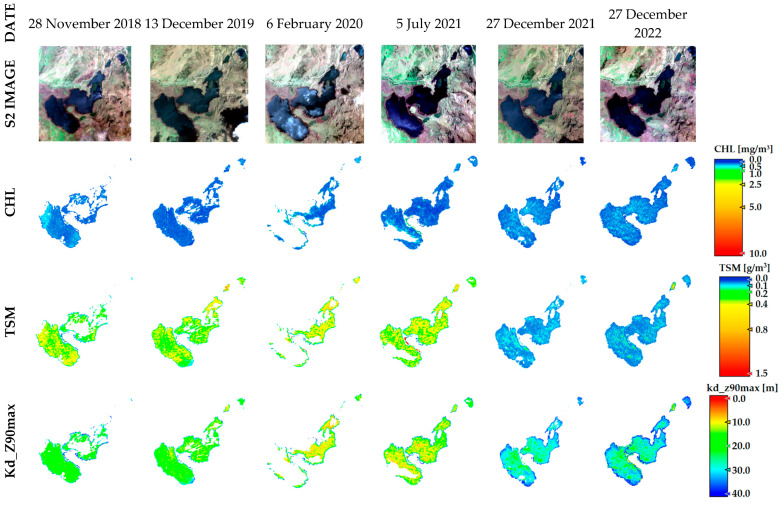
Multitemporal variability of CHL, TSM, and Kd_Z90max in Atillo lakes from 2018 to 2022 years.

The Colta lake is characterized by high concentrations of CHL with higher concentrations in the north zone. Nevertheless, this situation changed in 2020 as the CHL decreased considerably. This anomalous behavior is in line with the increase in Kd_Z90max, and this situation will be discussed in the next section (see Figure 8). The mean values of TSM showed a relatively stable behavior over time. Higher Kd_Z90max in Colta can be observed in February 2020 (Figure 6b), which is in concordance with a reduction in CHL values. High Andean zones are characterized by high humidity and persistent drizzle. Figure 6d shows a temporal profile with more precipitation than subsequent months. Therefore, apparently the precipitation did not necessarily cause high sediment discharges for these zones. This would be related to an increase in the water level from precipitation sources, and hence apparently the transparency is increased due to the optical conditions due to the passage of sunlight. The Atillo lakes presented the lowest values of CHL and TSM; these lakes are located within the protected zone of Sangay National Park in Chimborazo province, and anthropic activity is low. Therefore, the results are in line with high water quality. Medium CHL values range from 0.1 to 0.25 mg/m^3^ and TSM values range from 0.05 to 0.44 g/m^3^ indicating low variability considering the range of values to evaluate the trophic state of lakes [50] (see Figure 6c and Figure 9). Conversely, results for Kd_Z90max parameters show the capacity to introduce light into the water ranging from 13 and 33 m.

## 4. Discussion

The reflectance of the water bodies is overall low, yet most pronounced between 0.4 and 1.2 μm [51]. Within this spectral region, the reflectance signal is also easily affected by a high atmospheric influence, making it difficult to retrieve the pure water-leaving signal [18]. The most pronounced reflectance of clear water occurs in the blue, reducing towards the red and being zero in the infrared [52]. Hence, to evaluate water using optical sensors is possible due to the biophysical and chemical variables of water that produce an effect on its optical properties; this is used to study lakes, ponds, and other water bodies, and their respective changes over time. Water coloration changes were observed on RGB from S2-L1C images (see Figure 7) and the C2RCC versions made it possible to mark their changes and spatial distribution in detail. The water quality has a relationship with its color, and the differences of the reflectance spectrum are related to the dominant wavelength and water quality variables, such as the Secchi depth and CHL, among others [13]. The Yambo lake has a green coloration, which can be linked with minerals dissolved from the geology composition of the lake but also due to chemical characteristics (i.e., pH and P, among others) that suggest a high concentration of organic matter likely due to tourist activity wastes. S2 satellite information and its analysis enabled the detection of an abrupt change in water composition, evidenced by in situ sampling with the C2X-COMPLEX atmospheric correction version. Higher CHL values were observed and were according to the in situ monitoring data for Yambo lake. Also, an additional laboratory analysis with in situ water sampling showed in January 2021 a mean of 10.4 mg/L of nitrates, which agrees with the results identified with S2.

For the aforementioned variables linking to the trophic state is an important role for water studied with remote sensing. This study was based on three lakes with different conditions of eutrophication and conditions of water quality (i.e., Yambo: eutrophic [53]; Colta: eutrophic and mesotrophic [53]; Atillo: oligotrophic [54]). Comparing the data from in situ sampling with each version to water atmospheric correction for the studied lakes, the results showed that the C2X-COMPLEX was effective for Yambo and Colta. Although C2X is developed for waters with high concentrations of suspended material and CHL, the results using the C2X-COMPLEX method showed lower RMSE, BIAS, and MAPE% values. This method is developed for optically complex water types and inland waters. That this method is best suited can be attributed to the fact that Yambo and Colta have high levels of water contamination, anthropic activities are common in these places, and also Colta lake is subject to dredging processes; consequently, the water is continuously subject to change. According to Carlson [50,55], the mean values of CHL and Kd_Z90max suggest that the trophic state of Yambo is hyper-eutrophic in the monitoring date of 2021. The multitemporal analysis indicates that this eutrophication situation for Yambo was changing in the last few years where the lake changed to a eutrophic state. CHL values vary between relatively high values, which reveal a hyper-eutrophic state (>75 mg/m^3^), and low CHL values that reveal a eutrophic state (25 to 75 mg/m^3^). The Colta lake is eutrophic during multitemporal analysis, but in 2020 the water conditions changed and a mesotrophic state was observed in the results.

Conversely, the applicability of the C2RCC method was successful and it was accurate for higher water transparency and lower CHL and TSM values for the group of lakes of Atillo where the atrophic activities are limited. This is according to the use of this method for eutrophic to mesotrophic water types [25]. Results for Atillo determined that Atillo is oligotrophic and even ultra-oligotrophic, which is maintained throughout the multitemporal analysis. According to [18], uncertainty for low CHL concentrations is determined with a large error if the TSM concentration is high, but with a smaller error under low TSM conditions, which is in favor of the results of the Atillo lakes. The values for CHL and TSM had a mean of 0.16 mg/m^3^ and 0.38 g/m^3^, respectively (see Table 3).

After using optical imagery for water analyses, we noted that weather conditions such as high cloudiness and wind, typical for high mountain areas in equatorial regions, can influence the transparency values of water, altering the optical signal retrieval. The phenomenon of wind was evidenced on the Atillo lake, where the water body revealed the reflection of waves, therefore showing specular properties (see Figure 9, RGB S2 image of 2020 and July 2021). In the same way, the wind presence is typical for highlands, and produces waves on the water surface, which are also observed on the image products. The C2RCC method makes it possible to identify anomalous results linking with cloud-risk flag; even so, it was possible only for extreme anomalous values and, therefore, the image processing for these particular study zones required an additional process to obtain statistical analysis based on pure water pixels.

The obtained results help to understand the dynamics of the eutrophication states in the lakes. Also, additional in situ monitoring within the temporal dates analyzed on this study obtained in 2022 for Yambo lake shows a mean CHL value of 15.4 mg/m^3^ [56] and for 2017 a mean CHL value of 82.6 mg/m^3^ [57], which is in agreement with our results (see Figure 6 and Figure 7). A follow-up research aims to focus on applying field radiometry. That will help us to develop our own calibration models for high Andean lakes and minimize the uncertainty on products derived from current atmospheric corrections.

Lake eutrophication is considered a serious environmental problem, and only a few studies have focused on lake eutrophication in terms of anthropogenic influences (such as sewage emissions and agricultural practices) [58]. This environmental situation can be researched with calibrating models based on remote sensing, reducing uncertainty and increasing the spatial scale of studies. The climatic conditions for Andean regions produce high cloud cover, which is a problem for finding a collection of satellite images with good quality, but also the wind alters the conditions in optical analyses over this type of lake [59]. This was more evident in Atillo.

Due to the atmospheric conditions typical of this study zone, it is difficult to obtain a large quantity of images of entire lakes. Pixels were affected first by clouds and their shadows, and second by the effects of sun glint or land adjacency [40]. Hence, the processes to mask out these pixels for the area of water to study the lake are reduced in some cases. Even so, the results can offer important information about the conservation state of lakes.

## 5. Conclusions

Using the automatic C2RCC water quality products as offered by SNAP, it was possible to analyze the eutrophication of three different lakes under study in the Andean region. The atmospheric correction has an effect on the retrieval of signals from the waters of bodies of water. Due to lakes having their own biophysical characteristics, reflectance can be altered by water composition like CHL, and this is important for applying an adequate method of atmospheric correction. In Yambo, CHL values of 79.6 ± 48 mg/m^3^ were obtained using S2 images and automatic biophysical products with the C2X-COMPLEX atmospheric correction version, which were validated using in situ samples and showed lower RMSE than the C2RCC and C2X versions. The eutrophication state determined for Yambo was between eutrophic and hyper-eutrophic. Even so, a reduction in CHL and TSM while Kd_Z90max increased in the multitemporal analysis was noted, revealing that the lake had a reduced eutrophication state in the last seven years. The C2X-COMPLEX version was also evaluated as the best method for Colta with values of CHL of 0.5 ± 1.8 mg/m^3^. Colta is a lake with high anthropogenic activities, and the variability of biophysical variables not only depends on a natural process, but also their variability is affected due to dragging processes, which alter in an abrupt way the levels of chlorophyll and sediments. After multitemporal analysis, a mesotrophic state was determined for Colta. Automatic water quality products enabled the detection of sectors of contamination, mainly in areas near highways and areas with human activities due to tourism and socioeconomic activities in the surroundings of the lakes.

The Atillo lake has superior water quality with high transparency and lower anthropogenic activities, as opposed to Yambo and Colta lakes. The C2RCC was determined as the best atmospheric correction method for these water conditions after a comparison with in situ results. With a mean CHL value of 0.16 ± 0.1 mg/m^3^ for Atillo, an oligotrophic and even ultra-oligotrophic state was determined, which has remained stable in the last few years.

This study revealed the high capacity of automatic products of C2RCC atmospheric correction versions to study the lakes of highlands, with the spatial resolution of S2 images making it possible to detect sources of discharge (i.e., nutrients, release of sediments, and other suspended materials such as pollutants, heavy metals, or organic substances), by abrupt changes in the spatial analysis of biophysical variables, useful for water studies and monitoring. The C2XCOMPLEX approach was evaluated as an attractive option to study water bodies with complex dynamics and variability of water composition that alter the optical water properties. The C2RCC method was found adequate for lakes with high transparency, typical for lakes in the highlands with great water quality. Even so, due to specular characteristics on this water body and also the weather conditions, a careful processing of the images was necessary to achieve satisfactory results based on pure water pixels.

The Ecuadorian Andean region is characterized by a high percentage of cloudiness, limiting the use of optical images with good conditions for water quality monitoring. However, climatic conditions have low annual variability in this mountain equatorial region, which allows for multitemporal studies even though less imagery is available than in other regions.

Automatic water products from S2 give information about the water quality facilitating the local and temporal evaluation of the lake’s eutrophic status and the link to its potential causes. Our results can be used to establish strategies for protection and conservation. A next step for mountain lakes is research focused in the definition of our own atmospheric correction model or the establishment of our own correction parameters applied to the C2RCC versions, all with extensive radiometric measures in situ. Finally, the results of this study give an alternative to study these lakes located in complex zones. This is in line with the increasing global concern about water resources and environmental conservation, research aimed at understanding and monitoring water quality in ecologically sensitive regions like the Andean mountains. In addition, these results help us to generate a research project and obtain a grant (ESPOCH-IDIPI-336), which is funding the study of high Andean lakes from June 2023 to December 2026. The project focuses on the analysis of in situ reflectance to obtain calibration parameters. Hence, this will allow us to minimize the uncertainty in studies of lakes in the high Andean region and mountain lakes in general.

## Figures and Tables

**Table 1:** S2 images used for each study lake for multitemporal analysis.

Lake	S2 Date (Day-Month-Year) Used	Tile Number	% CC ^1^	Platform
Yambo	06 June 2017	17MQU	23.9	S2-A
	12 April 2018		11.4	S2-A
	24 August 2020		7.6	S2-B
	31 January 2021		27.3	S2-B
	27 December 2021		33.0	S2-B
	18 September 2022		22.8	S2-A
Colta	06 June 2017	17MQU	23.9	S2-A
	07 January 2018		20.0	S2-B
	27 May 2019		28.9	S2-A
	06 February 2020		17.7	S2-B
	28 September 2021		17.2	S2-B
	08 September 2022		22.6	S2-A
Atillo	28 November 2018		38.6	S2-A
	13 December 2019	17MQT	32.2	S2-A
	06 February 2020		16.0	S2-B
	05 July 2021		5.4	S2-A
	27 December 2022		24.5	S2-A

^1^ Cloud cover (CC) percentage for the entire S2 image.

**Table 2:** Comparative RMSE, BIAS, and MAPE% results of the CHL or Kd_Z90max product for each lake after applying the different atmospheric versions.

Lake-Biophysical Variable	Atmospheric Correction C2RCC Version								
	C2RCC			C2X			C2X-COMPLEX		
	RMSE	BIAS	MAPE%	RMSE	BIAS	MAPE%	RMSE	BIAS	MAPE%
Yambo-CHL [mg/m^3^]	94.7	−93.9	97.6	35.1	−26.7	28.4	***18.6***	***−14.6***	***14.9***
Colta-SD-Kd_Z90max [m]	36.6	35.9	1716.6	20.5	18.5	874.4	***3.1***	***1.8***	***91.0***
Atillo-CHL [mg/m^3^]	***0.8***	***−0.6***	***58.7***	3.8	1.47	169.5	9.2	6.25	673.1

**Table 3:** Statistical values (mean value± standard deviation) of variables in the lakes calculated for the Figure 5 images.

Lake	Image Data	CHL (mg/m^3^)	TSM (g/m^3^)	Kd_Z90max (m)
YAMBO	31 January 2021	79.6 ± 48	9.2 ± 11.4	0.8 ± 0.6
COLTA	6 February 2020	0.5 ± 1.8	4.4 ± 13.4	5.3 ± 2.4
ATILLO	5 July 2021	0.16 ± 0.1	0.38 ± 4.0	13.0 ± 0.2

## Data Availability

Not applicable.

## Supplementary Materials

The following supporting information can be downloaded at: https://zenodo.org/records/10030630, Table S1: Detail of comparative RMSE results of the CHL or SD product for each lake after applying the different atmospheric corrections.
